# Biomechanical Basis for Bone Healing and Osseointegration of Implants in Sinus Grafts

**DOI:** 10.1111/cid.13424

**Published:** 2024-12-05

**Authors:** Claudio Stacchi, Benjamin R. Coyac, Jill A. Helms

**Affiliations:** ^1^ Department of Medical, Surgical and Health Sciences University of Trieste Trieste Italy; ^2^ Department of Oral Biology, Goldschleger School of Dental Medicine, Faculty of Medical and Health Sciences Tel Aviv University Tel Aviv Israel; ^3^ Department of Surgery, Stanford School of Medicine Stanford University Stanford California USA

**Keywords:** bone grafting, bone regeneration, maxillary sinus, sinus augmentation

## Abstract

A thorough comprehension of the mechanisms controlling new bone formation and implant osseointegration after maxillary sinus floor elevation is crucial for aligning our treatment choices with biological principles and enhancing clinical outcomes. The goal of bone regeneration in sinus lift procedures is to provide a sufficient amount of newly‐formed tissue to support implant osseointegration. However, it is still unclear whether there is a minimum quantity of vital bone within the newly‐formed tissue required for effective support, though it is generally assumed that vital bone is essential for this process. The source and integration of new bone in maxillary sinus floor elevation procedures remain debated. Most clinical studies suggest a paramount role for sinus floor and bony walls, with a centripetal pattern of new bone formation, while conflicting reports exist regarding the osteogenic role of the Schneiderian membrane. The influence of mechanical input on peri‐implant bone formation, mineralization, and maturation is significant, with bone remodeling regulated by mechanical strains generated during loading. Defining optimal loading for implants, particularly in sinus lift procedures, is challenging, as early loading may damage interfacial tissue, interfering with osteogenesis. Differences in osseointegration dynamics between native and augmented bone may arise from biological and mechanical factors, but also from patient‐specific factors which should be evaluated in treatment planning. Factors to consider include sinus anatomy, patient and site‐specific regenerative potential, and the selection of graft material that matches the osteogenic and mechanical requirements. Tailored approaches integrating patient‐specific considerations and refined implant strategies will enhance predictability and longevity of treatment.

**Summary**

**What Is Known**

- After maxillary sinus floor elevation, bone graft particles must be surrounded and connected to one another by a network of new vital bone, which is essential for implant osseointegration and bone remodeling at the implant interface.
- Optimizing the surgical approach critically depends on understanding the cellular source of new bone and the molecular signals and mechanical stimuli that guide its formation.

**What This Study Adds**

- The clinician must evaluate maxillary sinus anatomy and the patient's bone regeneration potential—considering factors like age, smoking, metabolic disease, and co‐morbidities—to determine the feasibility and more convenient surgical approach.
- Mechanical stability is crucial for successful bone regeneration and implant integration, necessitating proper force distribution, careful timing and modalities of implant loading, and a surgical technique that stabilizes the graft material to support bone regeneration and ensure clinical success.

## 1 Introduction

Sinus floor augmentation procedures have enabled implant placement in atrophic posterior maxillae compromised by sinus pneumatization and/or by severe alveolar ridge resorption following tooth extraction. Several approaches have been developed to adapt local anatomy, that is, lateral, transcrestal, or palatal access (historical references include [1, 2, 3]). Implants can be placed simultaneously with an autologous bone graft or bone substitutes, or implants can be placed after the grafted material consolidated; this decision depends largely on the expected primary stability of the implant [4, 5].

The timing of implant placement relative to graft consolidation is still a matter of considerable debate [6, 7, 8], in part because clinical, histological, and radiographic analyses sometimes contradict quantitative data from animal models. In cases where the implant is placed after graft consolidation, particles of the bone graft substitute must have become entrapped in new vital bone (consolidated) in order to provide biological stability to the implant. The necessary or “optimal” ratio of vital bone/bone graft substitute has not been defined, but it is assumed that vital bone is necessary for remodeling of the bone at the implant interface [9]. Clearly, data are still required to fill in these knowledge gaps and consequently guide timing of implant placement.

In sinus lift procedures, there are two sequences of osseointegration: the integration (consolidation) of bone substitute particles, and the integration of the implant in bone. The origin of new bone that entraps the grafted particles and creates a composite mass is debated. Clinical studies demonstrated that new bone formation after sinus augmentation is negatively influenced by the distance from the native bone walls [10, 11]. However, recent preclinical models shed light to the contribution of the maxillary internal periosteum to new bone formation [12]. In vitro studies have also implicated the Schneiderian membrane as a potential source of osteogenic cells [13, 14]. A recent systematic review covering 26 preclinical studies, however, argues against the osteogenic potential of an isolated Schneiderian membrane. Instead, the review concluded that available evidence does not support that the sinus membrane significantly contributes to new bone formation following maxillary sinus augmentation procedures [15].

Since the purpose of performing a sinus lift is to generate new bone, the cellular source of that new bone and the molecular signals and mechanical stimuli that direct its formation become critically important. When compared to a dental implant placed in a healed extraction site, implants placed after a sinus lift procedure have a more complicated interface. Specifically, the crestal part of the implant is typically anchored in the native alveolar bone, and in the osseous floor of the maxillary sinus while the rest of the implant is in contact with a composite material made of graft particles, medullary spaces, and mineralized new bone. The biomechanics of this unique interface are largely undefined but are critical for the success of the implant. Understanding how mechanical loading and molecular signaling affect bone remodeling and implant integration is paramount for optimizing treatment protocols and implant design.

## 2 Biologic Processes in Maxillary Bone Formation and Healing

### 2.1 Unique Anatomical Features of the Maxilla and Its Sinus

From an anatomical perspective, the maxilla has a structure typical of bones that endure compressive loads: similar to vertebral bodies, the bony anatomy of the maxilla is characterized by a network of trabeculae contained within thin cortical plates [16]. In most craniomaxillofacial (CMF) bones, the outer and inner cortical plates are separated by diploë (spongy, cancellous bone that lacks a marrow cavity) but, in the maxilla, diploë is absent. Instead, the inner and outer layers of the bone are separated by a large air cavity, the maxillary sinus [17]. The sinus serves multiple functions including lightening the weight of the head skeleton, supporting the immune defense of the nasal cavity, moistening and warming inhaled air, enhancing voice resonance, and acting as a cushioning area to protect critical structures in case of facial injury [18].

The maxillary sinus is created through a process called pneumatization. During embryonic development the maxillary bone exists as a solid structure without air‐filled spaces. During post‐natal maxillary skeletal growth, bone resorption by osteoclasts creates voids, which will eventually become the paranasal sinuses. Osteoblasts, in turn, line the air‐filled cavities with new bone, and contribute to the formation of the maxillary and nasal periosteum [19].

The process of pneumatization is not complete until the individual reaches skeletal maturity. Tooth extraction, especially in the posterior maxilla, can lead to further pneumatization of the maxillary sinus [20]. When combined with post‐extraction alveolar ridge resorption, in most severe cases the net result can manifest as a radiographic union between the sinus floor and the cortical of the alveolar bone crest. This dramatic reduction in vertical bone height, in turn, can complicate dental implant placement [21]. Some studies suggest that ridge preservation procedures may help reduce sinus pneumatization following tooth extraction [22, 23, 24].

### 2.2 Cellular and Molecular Mechanisms Involved in New Maxillary Bone Formation

In the maxilla and elsewhere in the CMF skeleton, the periosteum serves as a source of stem/progenitor cells [12, 25]. Although CMF periostea resemble long bone periostea, the proliferative and repair potential is significantly lower than in long bones. For example, using a rodent model, investigators found that a tunneling procedure performed on the tibia triggers a strong osteogenic response from the periosteum [26] and formation of a large bony callus [27]. The analogous tunneling procedure performed on the oral surface of the maxillary bone stimulates resorption first, followed by a more attenuated osteogenic response from the periosteum [27]. Unlike extra‐sinus surgeries, where mucosa can be distinguished from the periosteum [28], intra‐sinus surgeries are complicated by a thin Schneiderian membrane that is clinically indistinguishable from the periosteum lining the sinus.

After maxillary sinus floor elevation, new bone formation starts in the blood clot filling the sub‐antral space delimited by the elevated Schneiderian membrane. Vascularization is fundamental for supporting the metabolic demands of bone formation, providing oxygen, nutrients and waste removal, facilitating the migration and differentiation of bone progenitor cells, and delivering growth factors necessary for osteogenesis [5].

Blood clot is usually mechanically stabilized using grafting materials but an alternative graftless technique is also possible. This method requires the simultaneous placement of implants during sinus floor elevation procedure, allowing the fixtures to support the membrane and counteract the pressure exerted on it during breathing [29, 30]. The limitation of this technique, which has also been validated by long‐term studies [31], is the need for an adequate amount of native bone to initially stabilize the implant. Furthermore, the vertical bone gain after healing will typically be less than the endosinus length of the inserted implants. A recent meta‐analysis suggests using implants longer than 13 mm to optimize the clinical success of this technique [32].

Biomaterials used in maxillary sinus floor elevation include autologous bone graft (autograft), bone‐derived materials (allografts and xenografts), synthetic materials, or combinations of the above. Autografts contain a mineralized matrix scaffold, growth factors, and mitotically active osteoprogenitor cells [33, 34, 35], which help generating new bone sooner than bone‐derived materials [36, 37]. Moreover, autogenous bone grafts offer the advantage of rapid angiogenic growth from surrounding host bone, facilitating vascularization of the graft and its cells. This process supports local metabolism, osteoclastic resorption, and functionally oriented osteoblastic remodeling. Resorption of autogenous bone releases growth factors like platelet‐derived growth factor and transforming growth factor ß, promoting stem cell proliferation, and activates macrophages, aiding in the formation of new capillary sprouts [38]. There are, however, notable limitations to autograft use for sinus augmentation: a finite volume of autograft can be harvested from any one site, which can be inadequate for reconstructive purposes. Autografts also tend to resorb over time, resulting in limited volumetric stability of the grafted area during consolidation [12, 39].

Non‐autogenous grafts (NAG) address both of these autograft limitations: an unrestricted quantity of the material is available, and NAG generally undergo limited resorption [12, 40, 41, 42]. NAGs, however, lack cells and are deproteinized if they arise from a xenogeneic source; both these features remove biological agents that promote bone formation [43, 44, 45]. Thus, in animal models directly comparing sinus floor augmentation, NAGs consolidate significantly slower than autografts [12]. On the other hand, NAGs resist resorption, which autografts undergo on a predictable basis. At present, no single material stands out as superior to the others in every clinical situation.

Barrier membranes (both resorbable or non‐resorbable) may be used to cover the antrostomy after performing lateral sinus augmentation. The mechanism of action of these membranes is to exclude epithelial downgrowth during healing and prevent graft displacement out of the sinus, especially if they have been fixed to the bone with pins. This displacement can occur during the early phases of healing if there is a sudden increase in endo‐sinus pressure [46]. Thus far, clinical trials and meta‐analyses have found no significant difference in implant treatment outcomes after sinus floor elevation with or without barrier membrane coverage of the lateral window (reviewed in [47]). There are contradictory studies, however, that suggest that barrier membrane coverage can increase new bone formation and implant survival [47]. Thus, there is a lack of agreement regarding whether covering the osseous window with barrier membrane enhances new bone formation and implant survival rates.

### 2.3 Role of Growth Factors, Cytokines, and Signaling Pathways in Bone Repair

**FIGURE 1:**
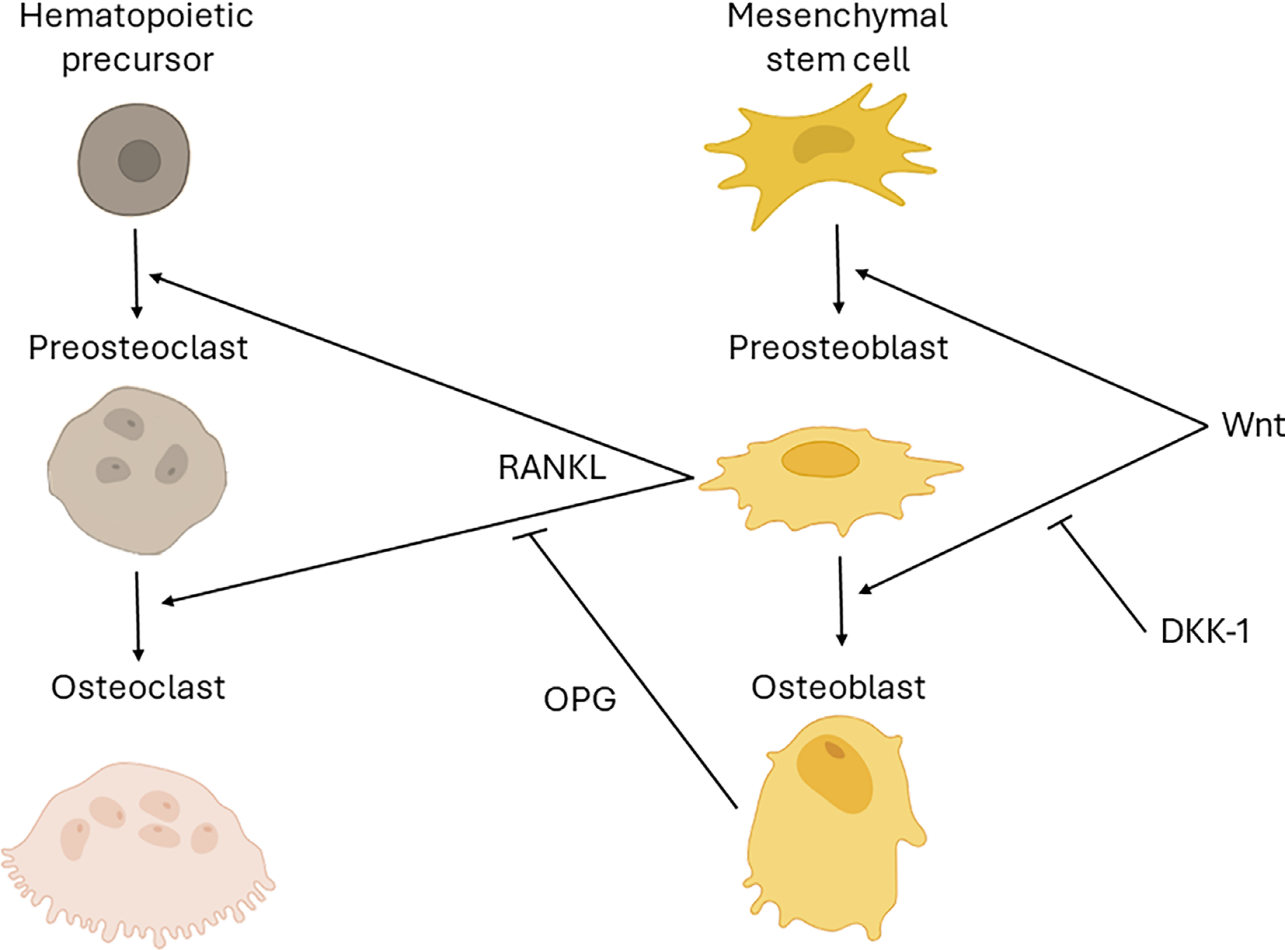
The Wnt signaling pathway plays a dual role in regulating the differentiation of both osteoblasts and osteoclasts. This pathway guides mesenchymal stem cells towards osteoblast differentiation. However, the DKK‐1 protein binds to the Wnt receptor complex on the surface of pre‐osteoblasts, inhibiting Wnt signaling and subsequently halting the proliferation and differentiation of osteoblasts. In their early stages, osteoblasts promote bone resorption by enhancing RANKL‐induced osteoclastogenesis. Blocking DKK‐1 enables osteoblast differentiation to proceed. Moreover, activation of the Wnt signaling pathway in mature osteoblasts increases the production of OPG, which inhibits RANKL‐induced osteoclastogenesis, thereby reducing bone resorption.

In addition to the aforementioned strategies involving grafting with autologous, allogeneic, xenogeneic, or alloplastic materials, new strategies are being developed that utilize biological factors for example, growth factors, enamel matrix derivatives, platelet‐rich fibrin matrix to hasten new bone formation. In some cases, combinations of bone substitutes and biological factors are being tested, with encouraging results [12]. One such growth factor is a member of the secreted WNT protein family (Figure 1). WNT3A protein is found in native bone [48] and in autografts [33, 49]. When recombinant WNT3A protein is added to autologous bone graft, it increases its bone‐forming capacity [36, 49, 50] via its ability to stimulate the proliferation of osteoprogenitor cells [36].

In general, when autografts are positioned in a site, a crucial initial phase involves the engraftment and survival of the cells present in the grafted material. Engraftment and survival phase are essential for autografts but does not apply to allogeneic and xenogeneic grafts, which lack viable cells. After engraftment, the surviving cells must begin to express osteogenic proteins and then differentiate into osteoblasts that secrete a matrix that contributes directly to new bone formation [49]. In the case of NAGs, the cells that populate the scaffold arise from tissues adjacent to the graft, for example, bone walls, membrane periosteum [51]. This step is rate‐limiting in bone grafting because both cell migration and cell differentiation are generally passive processes, unless a growth factor is employed [6, 12].

These preceding phases reliably occur when the autograft is harvested from a young subject. When the autograft is harvested from an aged individual, osteogenic gene expression of engrafted cells is significantly reduced [50]. All subsequent steps, where cells differentiate into osteoblasts and secrete an osteoid matrix to heal the defect, are also reduced if the donor is elderly [49, 50, 52]. The cause for this age‐related decline in osteogenic differentiation appears to be linked to a decline in endogenous Wnt signaling [53, 54]. Indirect evidence also supports that when Wnt signaling is reduced, because of elevated levels of the Wnt inhibitor, Sclerostin, the result is osteoporotic bone [55, 56].

NAGs do not suffer from an age‐related decline in efficacy, but they are complicated by the fact that there is a documented age‐related deterioration in the number and/or function of stem/osteoprogenitor cells in the host [57, 58, 59]. In the maxillary sinus, osteoprogenitor cells primarily reside in the bony walls and membrane periosteum, which can both be sites of new bone formation, at different rates. When a NAG is placed onto the osseous floor of the maxillary sinus, Runx2‐positive osteoprogenitor cells are activated and eventually encase the bone substitute with new bone. Some data suggest that patients with osteoporosis [60] and osteonecrosis have fewer [61] and/or less active [62] osteoprogenitor cells compared to healthy control groups (reviewed in [63]). NAGs are slower to consolidate relative to young, healthy patients due to this age‐related decline in bone‐forming capacity, and the fact that they are devoid of pro‐osteogenic proteins [64, 65]. This delayed graft consolidation contributes to greater variability in clinical outcomes when bone substitutes are used [66]. From a clinical standpoint, studies conducted on large patient cohorts confirm that patient age is a significant negative prognostic factor for the success of implants placed following maxillary sinus lift procedures [67, 68, 69].

Recombinant human bone morphogenetic protein‐2 (rhBMP‐2) has also been extensively tested in the field of sinus floor elevation. Two meta‐analyses found that using rhBMP‐2 in maxillary sinus floor elevation yielded clinical and histomorphometric outcomes similar to, or even less favorable than those of conventional sinus grafting procedures after a healing period of 6–9 months [70, 71]. Additionally, clinicians should consider the high cost of rhBMP‐2 and the fact that its use is not permitted in all countries.

## 3 Biomechanics of Osseointegration in Implants Inserted After Sinus Grafting

### 3.1 Mechanical Interactions Between Implants and Regenerated Bone in the Augmented Sinus

The roles of mechanical input in peri‐implant bone formation and mineralization should not be overlooked. In general, the regulation of bone remodeling for example, the coordinated formation and resorption of osseous tissue is influenced by mechanical strains produced in the bone in response to loading. The basic multicellular unit (BMU) is defined as a group of cells responsible for the process of bone remodeling, and consists of bone‐forming osteoblasts, bone‐resorbing osteoclasts, bone‐lining cells and osteocytes that reside within the bone matrix [72]. These cells interact both simultaneously and at various stages of differentiation with their progenitors, other cells, and the components of the bone matrix.

**FIGURE 2:**
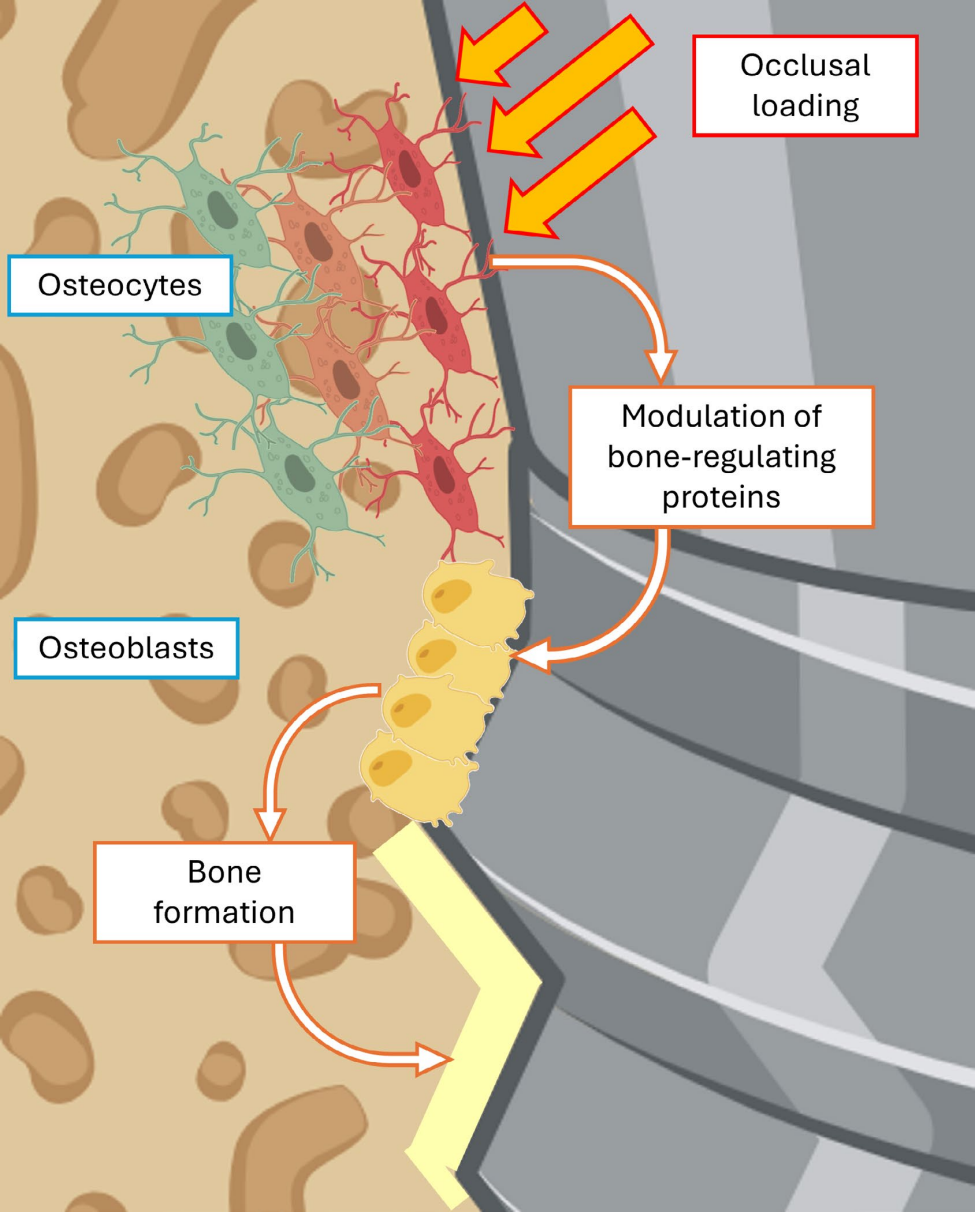
Mechanical loading of bone increases interstitial fluid pressure, creating fluid shear stress that deforms the cytoskeleton of bone‐embedded osteocytes, which modulate the release of bone‐regulating proteins such as RANKL, OPG, and sclerostin. Typically, mechanical loading activates osteocytes, which in turn enhance osteoblast activity, thereby stimulating bone formation.

The mechanoresponsive nature of the BMU helps ensure that bone adapts to changes in mechanical demands, maintaining bone strength and structure. When mechanical forces are applied to bone for example, during mastication, osteocytes detect these physical changes and signal to osteoblasts and osteoclasts to adjust their activity accordingly. The deformation of the osteocyte cytoskeleton triggers many different classes of mechano‐transducers which promote the release of bone‐regulating proteins (mainly RANKL, OPG and sclerostin) [73]. In general, mechanical loading applied to bone stimulates osteocytes, which in turn stimulate osteoblast activity, leading to bone formation (Figure 2). Conversely, reduced loading or disuse of teeth can lead to decreased osteocyte activity, which in turn causes increased osteoclast activity and bone resorption [74, 75].

Defining the appropriate loading regimen for an implant is challenging, especially in a sinus lift procedure. The new bone forming around an implant placed in an augmented sinus has a yield strength far below that of mature, healthy bone [74, 75]; consequently, even loading that is within some “physiological range” can trigger damage, which activates osteoclasts and bone resorption. For example, if an implant is subjected to loading prior to graft consolidation, data from animal models suggest that the interfacial tissue comprised of a blood clot and collagen may be subjected to excessive strains, which can interfere with osteogenesis [76, 77]. At this stage, in vivo and in silico models demonstrate that implant loading leads to deformation of the non‐consolidated tissue and displacement of graft particles away from the implant surface [6]. This displacement occurs regardless of the nature (e.g., autograft, NAG) of the grafted material.

Once the graft has undergone consolidation, the same mechanical loading of the implant results in lower strains. These strains are distributed throughout the new bone, or the composite particles of non‐vital hydroxyapatite meshed together by new bone. Finite element modeling demonstrates that at this stage, either type of material resists displacement, and therefore can effectively support an implant [6, 12].

The major mechanobiological difference between the response of an autograft vs. NAG appears to be that in response to loading, greater bone resorption occurs within an autograft [78]. NAG particles, on the other hand, tend to resist resorption [6]. Most NAGs, especially if thermally processed at high temperatures, are not resorbed due to their composition primarily of mineral components, like hydroxyapatite, which are highly stable and resistant to enzymatic degradation. Unlike organic bone components that can be broken down and replaced by new bone during remodeling phase, the mineral structure of NAGs remains largely unchanged over time. The apparent insensitivity of NAGs to resorption may be also due to the lack of vital osteocytes in the material. Normally, osteocytes are connected to one another via a canalicular network that is critical for the transduction of mechanical stimuli [79]. In non‐vital hydroxyapatite particles, osteocytes are absent: however, osteocytes populate the new bone that forms and connects the NAG particles together. This factor may contribute to consolidated NAGs being less sensitive to mechanical stimuli, resulting in reduced bone remodeling.

### 3.2 Biological Interactions Between Implants and Regenerated Bone in the Augmented Sinus

Osseointegration of an implant placed after autograft consolidation is similar to osseointegration in any healed site of the alveolar bone. At time of implant placement, mechanical primary stability is provided by the misfit between the implant external diameter and the radius of the osteotomy canal. Later, woven bone apposition in bone‐implant gaps followed by secondary remodeling of this newly‐formed tissue provides secondary stability within the augmented area.

In contrast, an implant inserted in a NAG‐augmented sinus is in contact with a heterogenous interface, that is, new vital bone (representing ~30% of the augmented area) together with particles of NAG (representing ~20% of the augmented area) [80, 81]. The presence of NAG along the implant surface does not significantly affect implant stability, as long as the sinus augmentation is consolidated [82]. Data is lacking regarding the dynamics of osseointegration of dental implants in contact with composite materials: however, in histomorphometric analyses of human tissues, the region of the implant in contact with NAG has a lower bone‐implant contact than does the part of the implant placed in native bone [83, 84]. This difference in BIC has been ascribed to a lower regenerative potential of the composite material compared to native bone [83, 84]. Authors speculated that differences are due to a lack of stem/progenitor cells in composite augmented bone compared to native bone [33, 49], but it may also be due to undefined changes in the blood clot that forms in native bone versus composite augmented bone.

### 3.3 Factors Influencing the Success of Implant Osseointegration in a Grafted Sinus

A series of factors have a direct impact either on the mechanical stability or on bone remodeling necessary for osseointegration in an augmented sinus floor. The dynamics of osseointegration around implants placed in native alveolar bone versus augmented bone in the sinus show some important differences:

First, provided there is no penetration of the Schneiderian membrane, the sinus is distinguished from other oral implant sites in that it constitutes a largely sterile environment [85]. This differs from implants placed in native bone where, despite best efforts, osteotomies are invariably populated by oral bacteria [86].

Second, unlike osteotomy site preparation procedures in pristine alveolar bone that typically involve copious flushing/irrigation of the site prior to implant placement [87], the sinus allows for retention of osseous coagulum that is generated during site preparation for a sinus implant [36].

Third, unlike other oral bone grafting procedures, sinus floor augmentation prior to implant placement allows for new bone formation in the absence of mechanical loading [74, 75].

Fourth, the shape of the maxillary bone provides an osteogenic space that supports new bone formation on multiple sides, that is, anterior, inferior, medial, and lateral bony walls [5].

Fifth, some studies have reported radiographic voids in an augmented sinus site, possibly due to insufficient compaction at the time of graft placement [88, 89]. Nonetheless, the majority of reports show that implant success remains high despite the presence of radiographic voids, and further, that higher bone density does not necessarily correlate with improved outcomes after 1 year of functional loading [90].

Sixth, the height of residual native bone crest (RBH) only moderately correlates with the overall bone gain after sinus lift procedure [91, 92, 93]. In addition, reduced RBH does not significantly hamper successful osseointegration within the augmented sinus [51, 94, 95, 96].

### 3.4 Role of Implant Design and Surface Characteristics in Biomechanical Stability

As previously seen, the newly‐formed tissue after maxillary sinus floor elevation is characterized by significant variability influenced by the effectiveness of the osteoconductive and potentially osteoinductive action of the biomaterial, combined with patient's general and site‐specific regenerative potential. In any case, the regenerated tissue can be generally considered as a low‐density bone tissue, with well‐represented marrow spaces and often characterized by the presence of residual graft particles. For this reason, when choosing the type of implant to use, a macrogeometry suitable for achieving good primary stability in low‐quality bone should be preferred. Therefore, if possible, it seems reasonable to choose tapered implants with deep threads, small pitch, and decreased helix angle [97], all characteristics that help improving primary implant stability in soft bone. Furthermore, for the same reason, it is appropriate to use implants with a large diameter, when compatible with the available thickness of the residual bone crest [98], but without exceeding 11–13 mm in length. In fact, as previously described, the percentage of newly‐formed bone in the augmented area follows a progressively decreasing gradient as we move away from the floor and the bony walls of the sinus [99, 100]. In particular, the vertical gradient of bone graft consolidation is steeper in the molar region, where the sinus cavity is usually wider: a recent human histomorphometric study showed that mean vital bone percentage decreased from 18.7% in the zone adjacent to the sinus floor to 12.8% at a distance of 8 mm [11].

The surface characteristics of the fixture also play an important role. Histologic and clinical evidence suggest that the establishment of a more favorable bone‐to‐implant interface occurs on rough‐surfaced implants compared to machined implants, especially in cases of poor‐quality bone [101]. Consequently, it can be expected that, when inserted into grafted bone, implants with a rough surface will exhibit better clinical outcomes than those with a machined surface. This hypothesis aligns with the findings of a review from Del Fabbro et al. [102], where machined implants inserted into grafted sinuses exhibited an average survival rate of 86.3% (involving 950 patients and 3346 placed implants), whereas implants with a rough surface demonstrated a mean survival rate of 96.7% (involving 2544 patients and 8303 placed implants). Moderately rough (Sa varying between 1.0 and 2.0 μm) [103] should be preferred to rough surface (Sa > 2.0 μm) as they both provide enhanced osseointegration, but the latter also represents an ideal substrate for bacterial biofilm adhesion and proliferation in case of accidental exposure in the oral cavity [104].

## 4 Clinical Implications and Future Directions

The successful consolidation of a graft depends on the gradual deposition of newly‐formed bone, subsequently leading to functional remodeling and, ideally, to the gradual substitution of the graft material with new bone. Together with patient's age, systemic conditions, and specific medication intake, the potential for regeneration is also determined by the local anatomy (large/narrow) of the sinus [5]. The procedure involves elevating the sinus membrane and exposing the endosinusal bony walls (typically the inferior aspects of the anterior, lateral, and medial walls, and the sinus floor). The front of new bone formation differs in multiple studies that addressed this parameter [12, 99, 105, 106].

There is a diversity of results when it comes to identifying the dynamics of new bone in a sinus lift procedure. For example, using xenogeneic deproteinized bovine bone combined with decalcified, freeze‐dried human bone matrix in patients, Hanisch et al. [106] showed that new bone forms evenly throughout the sinus space, without any differences according to the position in the sinus. Using xenogeneic deproteinized bovine bone particles in a murine model, Coyac et al. [12] demonstrated that the front of new bone formation proceeds centripetally from the periphery towards the center of the cavity. However, using autologous bone graft in a mouse model, Coyac demonstrated that new bone formed evenly in the sinus space, due to the presence of stem/progenitor cells in the autograft [12]. Histomorphometric analyses from this study revealed that approximately 30% of the new bone originated from cells present in the autograft. Still other authors have reported a centripetal new bone formation when using bio‐inert materials [99, 105] (Table 1). It seems reasonable, therefore, to hypothesize, despite the diversity of reported results, a general centripetal pattern of bone regeneration (from the floor of the sinus and walls towards the center of the cavity), modifiable by the use of grafts with osteogenic properties (e.g., autologous bone), whose engrafted cells can generate additional centers of ossification within the sinus cavity. The primary role of native bone walls to new bone formation in the sinus lift procedure is indirectly confirmed by clinical studies with lateral sinus augmentation. For example, Avila‐Ortiz et al. demonstrated that vertical bone gain and vital bone percentage in the regenerated area after 6 months of healing are negatively correlated with the size of the bony window [107].

**TABLE 1:** Dynamics of new bone formation after maxillary sinus floor elevation.

Study	Bone graft	Experimental model	Orientation of new bone formation
Hanisch et al. [106]	Xenogeneic bovine demineralized particles + allogeneic demineralized particles (DFDBA)	Human patients	Evenly spread throughout the sub‐antral area
Busenlechner et al. [105]	Anorganic bovine bone/deproteinized bovine bone mineral/aqueous paste of synthetic nano‐hydroxyapatite	Minipig preclinical model	Centripetal: new bone originating from the maxillary host bone and guided by the osteoconductive properties of biomaterials up to the Schneiderian membrane
Kolerman et al. [99]	Freeze‐dried bone allograft (FDBA)	Human patients	Centripetal: new bone is formed in larger quantities close to the maxillary osseous walls and floor than in the core of the sub‐antral area
Coyac et al. [12]	Xenogeneic bovine deproteinized particles	Mouse preclinical model	Centripetal: new bone is formed in larger quantities close to the maxillary osseous walls and floor than in the core of the sub‐antral area

The width of the sinus cavity also impacts the potential for forming new bone. Under equal conditions, it is generally easier to obtain new bone in the regeneration area when the sinus cavity is narrow [51, 96, 108, 109, 110]. This hypothesis has been demonstrated by histomorphometric human studies, which highlight a statistically significant negative correlation between the amount of newly‐formed bone and the latero‐medial width of the sinus cavity, both using lateral [108, 109, 110] and crestal approaches [51, 96]. Additionally, Stacchi et al. [111] demonstrated that the amount of newly‐formed bone varies in different areas of the same maxillary sinus in the same patient: it has been observed that the percentage of newly‐formed bone is significantly higher at mesial sites (usually presenting a narrower sinus cavity and close proximity to the anterior wall) compared to more distal sites, where sinus cavity is usually wider [111].

**FIGURE 3:**
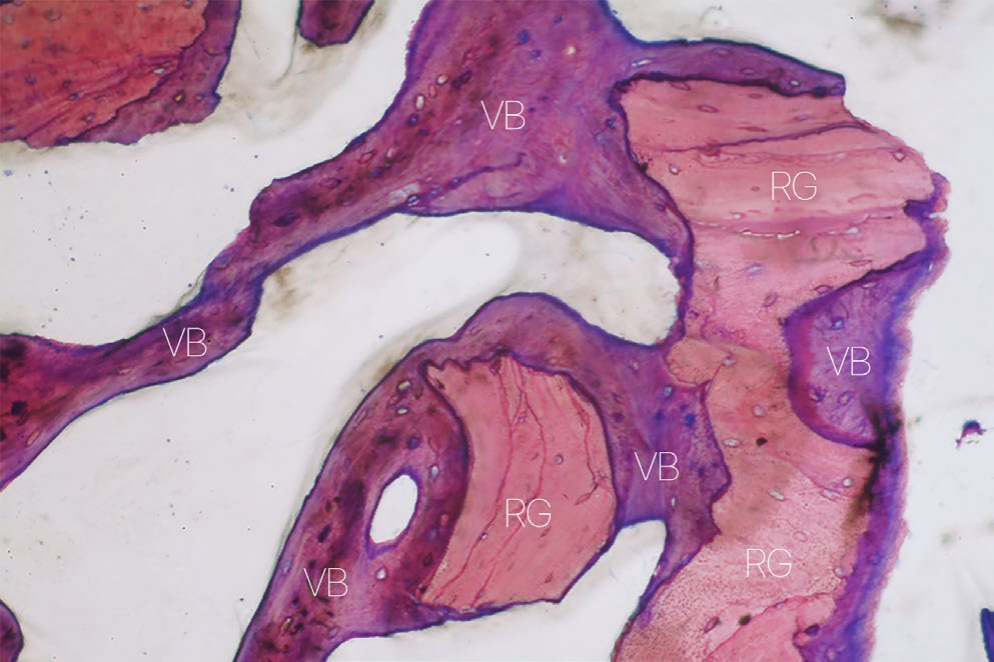
Histological specimen of regenerated tissue harvested 11 years after maxillary sinus floor elevation using a bovine‐derived bone substitute. The residual graft (RG) particles are still easily identifiable, surrounded and bridged by vital bone trabeculae (VB). Stained with toluidine blue and pyronin G, and observed at ×200 magnification.

A prolonged healing time allows newly‐formed bone to assume a more lamellar structure through remodeling; it does not, however, solve the problem of sites with low regenerative potential. Mean healing times generally vary from 6 to 9 months, where 6 months are preferred when using autogenous or allogeneic grafts and longer healing periods for xenogeneic and synthetic bone substitutes [112, 113]. It is worth noting, however, that numerous histomorphometric studies in patients showed that mean percentage of bone after sinus floor elevation performed with xenografts did not vary significantly between 5 and 15 months of healing [114, 115, 116]. A prospective study by Galindo‐Moreno et al. [117] showed that the percentages of new bone, residual xenograft particles, connective tissue, osteocytes, and osteoblasts were similar at 6 months, 3 years, and 7 years (Figure 3).

The fact that a wide sinus is an unfavorable environment for bone regeneration should be considered in the overall pre‐operative evaluation of the patient, when attempting to assess the predictability of the clinical outcomes of the procedure and plan possible corrective interventions (e.g., increasing healing time, more effective biomaterials, considering alternative therapeutic options). It must be clear that regenerative potential significantly varies among patients and in different anatomical situations. During treatment planning, it is important to inform patients presenting critical factors (smokers, systemic issues or medication intake, elderly, or large sinus cavities) about the lower predictability of regenerative outcomes in their specific case [118]. It should be considered the possible option of placing a short implant in native bone rather than a longer implant in combination with a sinus floor augmentation procedure, especially in patients with low regenerative potential. The posterior maxilla, characterized by soft bone quality, has long been regarded as the most challenging site for implant placement. Traditionally, implants placed in denser bone—more common in the mandible—have been associated with higher survival rates [119]. However, recent findings suggest that implants in the denser mandibular bone may be more vulnerable to masticatory forces, increasing long‐term risk of overload and aseptic implant failure [120, 121]. In fact, numerous recent meta‐analyses on short implants in the posterior maxilla confirm that, despite an unfavorable crown‐to‐implant ratio, their medium‐ and long‐term clinical outcomes are comparable to those of longer implants used in conjunction with maxillary sinus floor elevation [122, 123, 124, 125].

The significant variations in newly‐formed bone percentage, the continuous positive pressure exerted by the membrane on the regeneration area, potential inadequate graft compaction, and the varying volumetric stability of different biomaterials may contribute to shrinkage of the grafted area during the healing period, particularly in terms of height. Previous studies report contraction rates for both autogenous bone and bone substitutes ranging from 20% to 50% [126, 127, 128]. Graft volumetric shrinkage following sinus augmentation has been found to influence implant placement during two‐stage procedures, potentially affecting long‐term implant survival [128]. Ideally, the graft material would be completely resorbed and replaced by new bone formation, but in some instances, a long‐lasting scaffold is crucial to provide necessary support for osseointegrated implants [78]. The presence of a wide sinus with a large latero‐medial angle, and the loss of multiple teeth beneath the augmented sinus are strong predictors of significant graft resorption [129].

## 5 Conclusions

When planning implant‐supported rehabilitation of the atrophic posterior maxilla, it is important for the clinician to have a clear understanding of the biological and mechanical factors that influence bone regeneration after maxillary sinus floor elevation. First, the surgeon must carefully consider the quality and quantity of the residual bone crest and sinus anatomy in the area of interest for example, narrow vs. wide cavity to determine the feasibility and approach for the sinus lift procedure. Critical for this assessment is the inherent potential for the patient to regenerate bone. Age, smoking, metabolic disease, and underlying co‐morbidities should be carefully evaluated since it is this ability to generate new bone that will ultimately decide the success or failure of the procedure.

Second, achieving mechanical stability is vital for successful bone regeneration and implant integration. Proper distribution of forces on the implant‐ and the avoidance of overload‐ influence osseointegration, so the timing and modalities of implant loading are important variables. The surgical technique should provide adequate stabilization of the graft material used to lift the sinus membrane and support bone regeneration, as both are essential to preventing graft displacement and thus implant failure.

Finally, the clinician should carefully consider graft selection. Autografts, allografts, xenografts, and synthetic bone substitutes exhibit different levels of osteogenesis, and different mechanical properties, and when combined, yield a composite material with its own osteogenic and biomechanical properties. In this complex situation the chosen material should both provide and/or support osteogenesis while simultaneously resisting resorption. It behooves the surgeon to integrate these variables into a solution that will provide patients with predictable, long‐term implant stability.

## Conflicts of Interest

The authors declare no conflicts of interest.

## Data Availability

The data that support the findings of this study are available from the corresponding author upon reasonable request.
